# Suggestions to Derive Maximum Stocking Densities for Layer Pullets

**DOI:** 10.3390/ani9060348

**Published:** 2019-06-13

**Authors:** E. Tobias Krause, Lars Schrader

**Affiliations:** Institute of Animal Welfare and Animal Husbandry, Friedrich-Loeffler-Institut, Dörnbergstr. 25/27, 29223 Celle, Germany

**Keywords:** pullets, stocking density, broiler, welfare, housing, planimetric measurement, space requirement, poultry, rearing, modeling

## Abstract

**Simple Summary:**

The housing of farm animals, such as laying hens and broiler chickens, is regulated by the European Union (EU). However, for young laying hens which are not laying eggs yet, i.e., so-called pullets, no regulation for the number of birds per space is available. We exemplarily calculated maximum stocking densities for pullets based on their body size taking into account the European regulations for adult laying hens and broiler chickens. Our approach is mainly considering that a certain proportion of additional space should be provided to enable the birds to perform active behaviour.

**Abstract:**

Stocking densities for domestic chickens (*Gallus gallus domesticus*) are regulated by the Council Directives of the European Union for both laying hens and broiler chickens. For layer pullets no regulation of stocking density has been established yet. Based on the existing Council Directives for laying hens (1999/74/EC), broiler chickens (2007/43/EC) and calculations of the floor space that is required for the respective chicken’s body, we exemplarily calculated maximum stocking densities for layer pullets. Based on the calculations we obtained absolute additional spaces for birds of different live body mass classes, i.e., useable floor space that the birds have additionally available to the space covered by their body. This allowed us to calculate the relative additional space per individual. We suggest the relative additional space to be a key parameter to derive requirements for a maximum stocking density in layer pullets. We analysed several scenarios for pullets under consideration of the Council Directives for laying hens and for broiler chickens, coming to the conclusion that layer pullets at the end of their rearing period should be provided ideally with a relative additional space of about 40–60%.

## 1. Introduction

Maximum stocking densities for domestic chickens are regulated in the European Union (EU) by Directives of the European Council for both laying hens and chickens kept for meat production, to which we refer in the following as broiler chickens. Maximum stocking density for laying hens i.e., reproductive adult hens (usually from around 20 weeks of age on [1,2]), is regulated by Council Directive 1999/74/EC [3]. The Council Directive 2007/43/EC [4] regulates stocking densities for broiler chickens of both sexes. Broiler chickens are kept from the day of hatching usually until a maximum age of 6 weeks [2]. At first glance, the housing of domestic chickens seems to be comprehensively regulated. However, the period prior to laying in the respective young hens, so-called pullets, has been neglected by the legislation so far. The lack of legislative coverage for pullets results in pullets often being kept at high stocking densities. This early rearing of chickens is of specific importance (e.g., [5,6,7]) for the development and welfare of the birds and represents usually more than a quarter of the entire life span of a commercially housed laying chicken, which undergoes a laying cycle of usually up to 72–74 weeks [8,9]. Here, we suggest exemplary calculations about how a normative maximum stocking density for layer pullets might be defined. The aim of our exemplary calculations is to provide proxies for a maximum stocking density for layer pullets that are orientated at the amount of space the animal requires. The results may on the one hand be transferred into legal requirements for layer pullets and on the other hand, they should be feasible for farmers. We aimed to settle our exemplary calculations on the already formally existing normative regulations for the maximum stocking density of broiler chickens and laying hens [3,4] and on the area that is covered by the animal’s body. We, here, did not consider any space requirements that could be derived from specific behavioural performances of chickens [10,11,12,13], and also not strain-specific differences in behaviour between laying hen and broiler chicken strains [14,15,16]. We did this intentionally for reasons of simplicity and data availability, and, thus, we did not further include any ethological parameters or nearest-neighbour distances into our exemplary calculations. The existing European regulations for stocking densities for layers (1999/73/EC) and for broilers (2007/43/EC) were taken as the reasonable and normative basis for our calculations.

Interestingly, both Council Directives [3,4] define the maximum stocking density in different ways. In Council Directive 1999/74/EC for laying hens [3], number of birds per m^2^ usable area define the maximum stocking density, which must not exceed nine hens per m^2^ [3]. Defining a fixed number of birds per space for laying hens seems to be well suited as these birds have undergone the majority of their growth (Table 1, e.g., [9,17,18]). In contrast, broiler chickens are extremely fast growing during the time of their housing (Table 2, [19]). Consequently, in Council Directive 2007/43/EC [4], the maximum stocking densities are defined by the amount of live body mass (in kg) that can be housed per m^2^, which must not exceed 33 kg/m^2^, and 39 kg/m^2^ under specific conditions, or maximally 42 kg/m^2^ (see 2007/43/EC, article 3(2–5)).

In case that the body masses of the animals are known, the two parameters for stocking density (i.e., animals or mass per space) can be converted into each other. For example, broiler chickens with an average live body mass of 2.144 kg (see Table 2) can be housed under certain conditions with a maximum stocking density of 42 kg/m^2^ (2007/43/EC). This would mean that at the end of the fattening period a maximum of on average 19.59 broiler chickens could be housed per m^2^ (Equation (1a)). Similar calculations can be conducted vice versa for laying hens. Here, nine hens per m^2^ usable space are allowed. Thus, at the end of the production period hens with a mean body mass of e.g., 1.8 kg (Table 1) could be kept with an average stocking density of 16.2 kg live mass per m^2^ (Equation (1b)). 

Equation (1a): Number of chickens (*x*) that can be kept per m^2^ if the maximum stocking density (*StD_mass_* defined as live body mass per space; = y in Equation (1b)) and birds’ live body mass (*Z*) are known. In this example, the maximal *StD_mass_* is assumed as 42 kg/m^2^ and the average live body mass as *Z* = 2.144 kg.

(1a) $$x=\frac{StD_{mass}}{Z}=\frac{42\;\frac{kg}{m^{2}}}{2.144\;kg}$$

Equation (1b): Live body mass (*y*) that can be kept per m^2^ if the maximum stocking density (*StD_number_* defined as number of birds per space, = x in Equation (1a)) and average birds’ live body mass (*Z*) are known. In this example, *StD_number_* is assumed as 9 hens/m^2^ and the average live body mass is *Z* = 1.8 kg.

(1b) $$y=StD_{number}\times Z=9\frac{birds}{m^{2}}\times 1.8\;kg$$

Due to the possibility of converting the space requirement into the number of birds per m^2^ in body mass per m^2^ and vice versa, it was possible to use the existing legal normative requirements for both layers and broiler chickens as starting assumptions to exemplary calculate possible maximum stocking densities for layer pullets. Since layer pullets are also undergoing a steep growth during the first 20 weeks of life, it seemed justified to define a normative upper stocking density threshold by live mass per space, as it is the case for broiler chickens. On the other hand, after reaching maturity, they are regarded as laying hens and their respective maximum stocking density is defined from thereon by the number of individuals per area usable space. To propose a regulation of the stocking density for layer pullets, it was worth to consider as reference both the European regulation for laying hens (1999/74/EC) [3] and for broiler chickens (2007/43/EC) [4]. 

## 2. Exemplary Calculations for a Maximum Stocking Density for Layer Pullets

All animals including domestic chickens of any strain require a certain amount of floor space to be physically present in a stable. Animals of course do not only require floor space but also space in the third dimension. However, for our exemplary calculations, we focused on the two-dimensional floor space, specific height requirements need to be discussed elsewhere. 

The birds’ bodies cover a certain area of floor space due to their pure presence [23,24,25,26], which we refer to as “floor space requirement” (FSR). However, beside this pure FSR, the birds need additional space to perform behaviours e.g., to move, to feed, etc. [23,24,25,26]. FSR’s differ between different behaviours of chickens [23]. Sitting requires slightly more FSR than standing [23]. For our exemplary calculations, we considered the FSR’s of standing chickens for two reasons. First, most active behaviours are performed in a standing position such as locomotion, feeding, social behaviours, etc. Second, when chickens are sitting, they are mostly resting (except e.g., for dust bathing) and, thus, do not require as much additional space as active chickens. 

Additional space should be regarded as a key quality parameter for sufficient space of captive animals as the FSR represents only the necessary and immutable space for an individual to a given time point to be present. Thus, the additional space is the crucial parameter to estimate the quality of a given stocking density as it allows the animals to perform behaviours, and ideally their entire natural behavioural repertoire. The FSR of chickens (and other animals) are not fixed values, but increase with increasing body size and body mass of the individuals, which especially in young birds is strongly linked with age. However, in theory FSR does not increase linearly with body mass/size of the chickens, but slightly lowers. This is due to the fact that FSR is a parameter with a quadratic exponent (i.e., cm^2^) and the size of an individual is a parameter reflecting a volume and thus has a cubic exponent (i.e., cm^3^). Planimetric measurements in poultry often assume linear fits between FSR and body size/mass, which is often suitable; alternatively, non-linear relationships might also be explored as done, e.g., for domestic turkeys [27]. Based on the available data for floor space requirements of pullets, laying hens and broiler chickens, as well as their changes over the growth period [23,24,26], we calculated for layer pullets the FSR’s at different body masses. 

We assumed for the laying hens a maximum stocking density of nine hens per square meter usable space according to the Council Directive 1999/74/EC [3]. For broiler chickens we assumed the three available stocking densities that are allowed under certain prerequisites, i.e., 33 kg live mass per m^2^ (2007/43/EC; article 3.2), or 39 kg/m^2^ (2007/43/EC; article 3.4) or maximally 42 kg/m^2^ (2007/43/EC; article 3.5) [4]. In combination with the regulations for stocking densities for laying hens and broiler chickens (1999/74/EC and 2007/43/EC [3,4]) we calculated the respective additional space. As a result, we have a proxy for the additional space that should be provided to layer pullets. 

More specifically, in our exemplary calculations we compared how much floor space is provided to the birds corresponding to the above-mentioned Council Directives 1999/74/EC and 2007/43/EC [3,4]) and how much FSR was occupied by the birds’ bodies at a respective growth stage. From the difference of both measures we obtained the additional space (provided space minus FSR equals the additional space), which is available per bird for behavioural performances.

We considered the rearing period of layer pullets to last until 19 weeks of age and to about 1.6 kg of live body mass (see Table 1). In our exemplary calculations, we considered the FSR’s of standing chickens of the strains LT (Lohmann Tradition), LB (Lohmann Brown) and LSL (Lohmann Selected Leghorn) according to the FSR functions from Spindler et al. [23] (see Equations (2a–c)) in response to the live body mass (*x*). 

Equations (2): Functions of the relationship between FSRs (floor space requirements) of chickens at a given body mass (*x*) for three laying strains a–c, from [23].

(2a) $$FSR_{LT}=0.2302x+106.29$$

(2b) $$FSR_{LB}=0.2031x+124.2$$

(2c) $$FSR_{LSL}=0.2247x+88.811$$

We calculated the FSR stepwise for every 0.1 kg of live body mass for each of the three lines. For the further calculations, we took the average FSR of the three strains (i.e., FSR_x_), to simplify and to generalise our exemplary calculations (see Supplementary Material Table S1). The additional space under consideration of the Council Directive for laying hens (1999/74/EC) was calculated as follows. Nine hens per m^2^ usable space are allowed at maximum. Thus, each laying hen had a space of 1111.11 cm^2^ available (Equation (3)). 

Equation (3): Minimal amount of usable floor space available per hen (*x*) according to the Council Directive 1999/74/EC that nine hens per m^2^ usable space can be housed.

(3) $$x=\frac{1m2}{9\;hens}=10000\frac{cm2}{9\;hens}=1111.11\frac{cm2}{hen}$$

From the floor space available per bird in accordance to the Council Directive 1999/74/EC (Equation (3)), the average FSR_x_ for layer pullets (Table S1) was subtracted. The resulting difference represents the additional space available for pullets (see Table S2). As an example, for a layer pullet of 900 g live body mass under consideration of Council Directive 1999/74/EC, the following resulted: A pullet of 900 g has 1111.11 cm^2^ available space in the stable and its FSR is 303.8 cm^2^ (Table S1). Thus, the difference is roughly 807 cm^2^, which represents its additional space (compare Table S2). 

For broiler chickens, FSR values from the strain Ross308 were available for three different body mass stages [26], which we used to illustrate that a function for FSRs in relation to live body mass can be easily obtained based on few measures, which might be interesting for the consideration of other strains or breeds. By plotting the FSR values as a function of the body mass, we calculated the formula of this relationship, using Microsoft Excel 2016, assuming a linear relationship. The values from [26] were: (i) Ross308 broilers at 1.696 kg body mass had a FSR of 303.28 cm^2^; (ii) at 2.168 kg body mass a FSR of 342,67 cm^2^; and (iii) at 2.733 kg body mass a FSR of 410.77 cm^2^. This resulted in the following relation between FSR (*y*) and body mass (*x*) for broiler chickens at the example of Ross308 (Equation (4)), an alternative function is available from [28]. 

Equation (4): Functions of the relationship between FSRs (floor space requirements) of broiler chickens at a given live body mass (*x*) based on the data from [26]. 

(4) y=0.1042x+123.1

Using this formula (Equation (4)), the FSR for each live body mass stage was calculated for every cumulative 0.1 kg. Furthermore, for each live body mass stage, we calculated the stocking density according to Council Directive 2007/43/EC in three different variants: For a maximum of 33 kg/m^2^, 39 kg/m^2^ and 42 kg/m^2^ (2007/43/EC; article 3.2; 3.4; 3.5, respectively). The respective theoretically resulting stocking density [10,000 cm^2^ /number of chickens] was used to calculate the offered space per chicken for each of the three respective variants provided in the Council Directive 2007/43/EC. From the respective available space, the FSR at the different live body mass stages (Equation (4)) were subtracted. This resulted in the additional space per bird at each live body mass stage (Table S2) under these three broiler chickens’ scenarios. We illustrate these calculations by two examples and remark a theoretical pitfall at low live body mass stages, as here, in theory, stocking density can be very high, which is not usually the case in practice. The two examples for additional space calculations for broiler chickens are the following. 

Example A: Chickens with 600 g live body mass according to Equation (4) have a FSR of 185.62 cm^2^ per bird (0.1042 × 600 + 123.1 = 185.62). In accordance with Council Directive 2007/43/EC, a stocking density of 42 kg/m^2^ is allowed under certain circumstances. In a flock with broiler chicken of 600 g live body mass this would, in theory, result in up to 70 chickens per square meter (42,000 g/600 g = 70). This means that under such a scenario, each chicken is offered a space area of 142.86 cm^2^ per bird (10,000 cm^2^/70 = 142.86 cm^2^). The difference from offered space and the FSR of the birds at this live body mass stage (185.62 cm^2^) would result in a negative additional space per chicken (Table S2); 142.86 cm^2^ − 185.62 cm^2^ = −42.7 cm^2^). Thus, the birds would not have enough space for their bodies. However, as mentioned above, in practice, such high stocking densities of 70 birds per m^2^ will not occur, as farmers usually calculate the number of day old chickens housed in a stable based on the expected slaughter live body masses. 

Example B: Here, chickens with a live body mass of 1.6 kg are considered. Equation (4) shows that each bird has an FSR of 289.82 cm^2^ (0.1042 × 1600 + 123.1 = 289.82). Broiler chickens with a live body mass of 1.6 kg could be housed at a density of 26.25 birds per square meter (42,000 g / 1600 g= 26.25). This would mean that the space provided per birds is 380.95 cm^2^ / bird (10,000 cm^2^ / 26.25 = 380.95 cm^2^). The difference between offered space and FSR results in an absolute additional space per bird of 91.13 cm^2^ (380.95 cm^2^ − 289.82 cm^2^ = 91.1 cm^2^) (Table S2).

**Figure 1:**
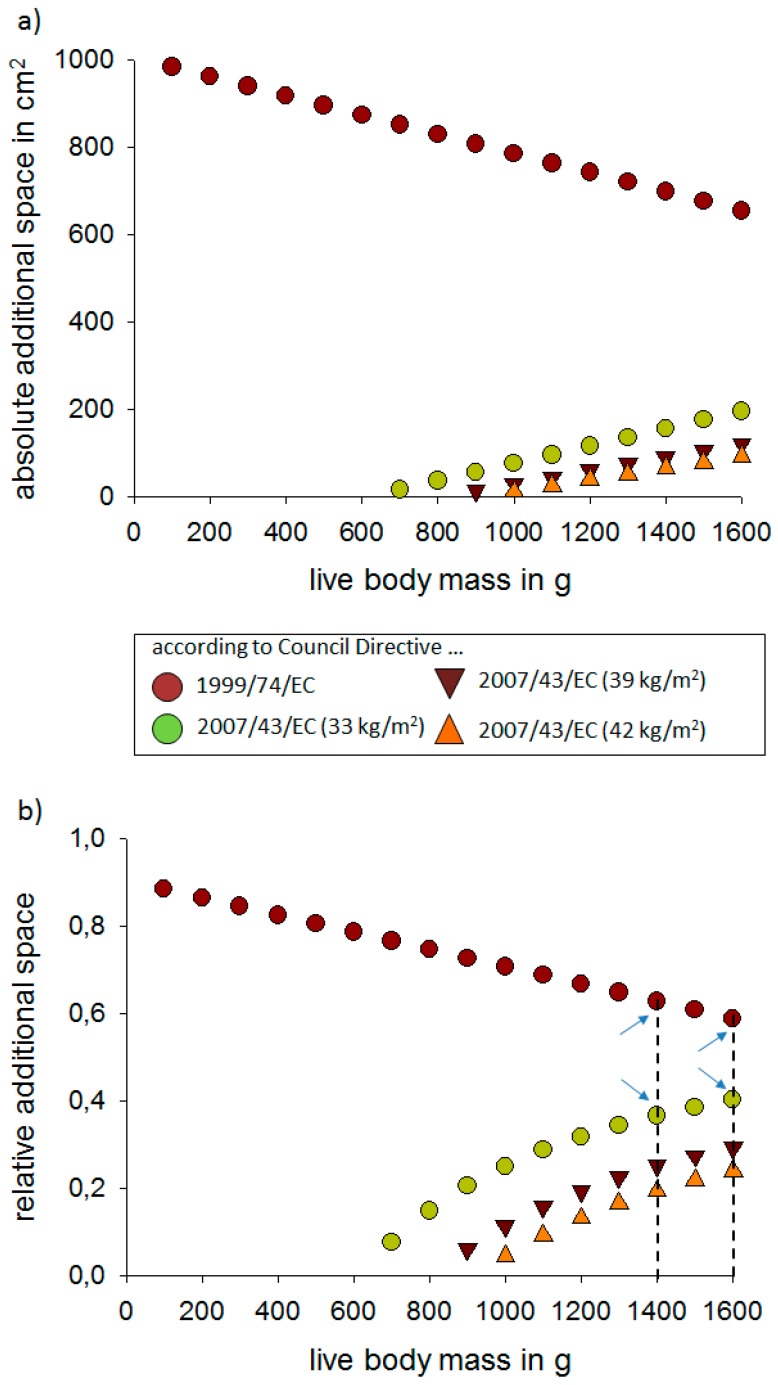
Outcome of the exemplary calculations. Amount of (**a**) the absolute additional space, and (**b**) the relative additional space that is available to a layer pullet of a given live body mass under the assumptions of the Council Directive 1999/74/EC, indicated by the red dots) for laying hens, the Council Directive 2007/43/EC for broiler chicken with either 33 kg/m^2^ (article 3.2; green dots), 39 kg/m^2^ (article 3.4; dark brown triangles), or 42 kg/m^2^ ( article 3.5; orange triangles). The dashed lines in (b) at *x* = 1400 g and *x* = 1600 g indicate typical live body masses when pullets reach maturity (compare Table 1 and Table 2). The blue arrows pinpoint the relative additional space at these live body mass stages.

The general results of our exemplary calculations showed two different patterns for the absolute additional space (Figure 1a). First, when we admitted layer pullets the same space as they would have as layer hens (1999/74/EC), they have relatively more absolute additional space early in the rearing phase which, however, decreases over time. The second pattern resulted from treating layer pullets similar to as broiler chickens (2007/43/EC). Early in life, the absolute additional space is limited (or even negative, see above; Table S2), but increases over time. However, at no point is the level of the layer-like variant reached. It is remarkable that the layer variant pattern does not reflect the birds’ development although it provides more absolute space throughout the rearing period of layer pullets. In contrast, the slope of the three broiler variants better match the birds’ development. Very early in life the absolute additional space required for the birds is smaller than later in life as with increasing age and increasing live body mass the absolute additional space should also increase. 

However, the additional space should reflect the need for increasing space with increasing age and live body mass of birds. Thus, instead of the absolute additional space it seems to be more appropriate to consider the relative additional space that is available per bird. In order to calculate the relative additional space per bird, the absolute calculated additional space (Table S2; Figure 1a) is divided by the sum of the total area per bird (i.e., FSR plus additional space). The outcome is shown in Table S3 and Figure 1b.

## 3. Discussion for Defining Legal Regulation at a European Level

As a result of our exemplary calculations and considerations, we recommend to regulate the maximum stocking density for layer pullets on the basis of the additional space, or, more precisely, on the relative additional space. From our perspective, the relative additional space is the key parameter enabling chickens to perform their behaviours. As shown in our calculations it is easy to calculate the FSR (i.e., the required floor space that hens cover with their body) in relation to the live body mass (this can also be converted to an age-dependent function, see [29]). However, the reliability of the calculated FSRs strongly depend on the availability and quality of data on the body area of birds [23,26]. Moreover, the FSR can significantly differ between chickens of different genetic lines (see Table S1, [23]). Such differences may be taken into account for defining any legal requirement for a maximum stocking density of layer pullets. 

In order to define a normative value for the maximum stocking density for layer pullets, a certain value for the relative additional space chickens that shall be provided to the birds during the rearing period must be specified. This value then results in a dynamic absolute additional space that increases in parallel to the growth of layer pullets. Thus, it is possible to calculate a respective maximum stocking density for each requested live body mass class. However, it is neither practical nor desired with respect to animal welfare that farmers continuously reduce the flock size with increasing live body mass of chickens in order to just not exceed the maximum stocking density, as thereby certain amounts of birds need to be removed from their familiar environment/group. 

With our exemplary calculations, we strictly follow a formal approach by using already existing normative regulations for the maximum stocking density of broiler chickens and layers. We, thus, do not consider any space requirements that can be derived from behavioural needs of chickens [30]. However, the existing requirements for the maximum stocking densities for broiler chickens and layers had been agreed in a political decision process in which both the behavioural needs of birds and the economic interest of farmers had been taken into account. Thus, the existing regulations for maximum stocking densities can be assumed as a normative basis for our exemplary calculations. 

Based on these considerations we suggest that layer pullets should be treated with respect to the stocking density between the legal requirements for laying hens (1999/74/EC) and for broiler chickens (2007/43/EC), as they are fast growing and will become laying hens. It is hard to justify that a layer pullet needs less space compared to a broiler chicken. With respect to the reduced activity of broiler chickens due to their fast growth, it is plausible that the more active and agile layer pullets require more floor space. On the other hand, the space offered to laying hens might be sufficient also for layer pullets. 

For the stocking density of layer pullets, the most relevant live body mass range probably is between 1.4–1.6 kg, thus, i.e., the live body mass when the pullets reach maturity (compare Table 1 and Table 2) and where space in rearing might be most limited. In this live body mass range, as highlighted in Figure 1b, the relative additional space ranges between about 60% when assuming space requirements for layers [3] and about 40% when assuming space requirements for broiler chickens in reference to Council Directive 2007/43/EC article 3.2 (33 kg/m^2^) or about at least 20% in reference to article 3.5 (42 kg/m^2^) [4]. In order to simplify the calculations, it is also possible to calculate the number of birds per square meter usable space based on the relative additional space. To give some examples, we did so for the relative additional space of 40% (see Equation (5a–g)). Calculations for 60% and 20% relative additional space are provided in the electronic supplement (see Equations (S1) and (S2)).

Equation (5): Example of a calculation to transfer a relative additional space of 40% into a stocking density with birds/m^2^.

(5a) $$rel.add.space\;\left(R\right)=\frac{additional\;space\;\left(Z\right)}{additional\;space\;\left(Z\right)+space\;covered\;by\;body\;\left(FSR\right)}$$

(5b) $$\;\frac{4}{10}=\frac{Z}{Z+FSR}$$

(5c) $$\frac{4}{10}Z+\frac{4}{10}FSR=Z$$

(5d) 4Z+4FSR=10Z

(5e) 4FSR=6Z

(5f) $$\frac{4}{6}FSR=Z$$

(5g) $$\frac{2}{3}\;FSR=Z$$

The calculations (Equations (5), (S1) and (S2)) reveal that in order to provide 40% relative additional space, for each pullet 2/3 of its FSR, i.e., the area covered by its body, needs to be additionally provided. In order to offer 60% relative additional space (Equation (S1)), each pullet requires 3/2 (1.5 fold) of its FSR area as additional space (for 20% relative additional space for each pullet ¼ of the FSR is required additionally). Thus, we could now calculate for certain chicken strains the stocking density under assumption of a relative additional space of 40/60%, based on the equations from [18], as indicated in Equations (2a–c). For LSL chickens, the producer provides a starting live body mass of laying hens from 1.385 kg. Under the 40% relative additional space scenario, this would result in a total area of about 666 cm^2^ (see Equation (6)) at the end of the rearing period of the layer pullets. When each bird is provided with that space, it is possible to house about 15 birds/m^2^ (i.e., 10,000 cm^2^/666.7 cm^2^ = 14.999).

Equation (6): FSRs of the LSL chickens (Equation (2c)) at the live body mass of *x* = 1.385 kg including the factor for a relative additional space of 40%, i.e., 2/3 (≈0.667), derived from Equation V for a. 

(6) $$FSR_{LSL+40\%rel}=\left(0.2247\times 1385+88.811\right)\times \left(1+0.667\right)=666.7cm^{2}$$

Calculating this for LSL chickens analogously for a relative additional space of 60% results in a maximum stocking density of 10 birds/m^2^ (i.e., 9.999 hens/m^2^). For heavier LB hens, which start as laying hens at about 1.65 kg (Table 1 and Table 2), the 40% relative additional space would result in a maximum stocking density of 13.06 birds/m^2^ at the end of the rearing period of the respective layer pullets, and for 60% relative additional space to a maximum pullet stocking density of 8.71 birds/m^2^. For only 20% relative additional space the maximum stocking density for LSL and LB would be 19.99 hens/m^2^ and 17.41 hens/m^2^, respectively.

A crucial aspect regarding the maximum stocking density is the available usable space in rearing systems for layer pullets. Often pullets are reared in multi-tier systems, which is recommended to adapt the pullets to the housing which they face as laying hens. Such multi-tier systems often are equipped with perches or grids at the edges of the tiers helping the birds to safely reach a certain tier or to move between tiers. In addition, these perches or grids are frequently used by the pullets for resting. In housing of laying hens, however, these perches or grids or not counted as useable area because according to the Council Directive 1999/74/EC, article 4(3) where it is stated that “the levels must be so arranged as to prevent droppings falling on the levels below”. Thus, only tier-levels equipped with manure belt can be regarded as useable area in layer stables. Manure belts reduces ammonia emissions from layer housing systems as they are used to regularly remove the droppings from the stables. This is meaningful in particular with respect to the long housing period of laying hens that may last longer than a year [8,9]. As layer pullets are kept for a comparable shorter period in the rearing stable it might be worthy to consider whether here the area above the perches or grids at the edges of tier-level can be counted as usable space.

## 4. Conclusions

The relative additional space in relation to the birds’ weight can be a useful parameter to derive requirements for the maximum stocking density in layer pullets. Our approach using exemplary calculations easily allows to calculate specific national legislative normative thresholds for stocking densities in layer pullets. Thus, the calculations provide a useful tool for political decisions. We analysed here a scenario for layer pullets under consideration of the Council Directives for laying hens (1999/74/EC) [3] and for broiler chickens (2007/43/EC) [4] and came to the conclusion that layer pullets at the end of their rearing period shall ideally be provided with a relative additional space of about 40–60% to treat layer pullets at least as well as broiler chickens (Figure 1b). Alternatively, when considering the number of birds per usable space on the basis of the relative additional space, this number should be for layer pullets, based in our exemplary calculations, between 9–15 birds per square meter usable space. 

Relative additional space provides a flexible measure for growing birds, such as layer pullets, but the same approach as used by our exemplary calculations can be used for other farm animals, where FSR values are available as for example in other poultry [26,27] or pigs [31,32,33,34].

## Figures and Tables

**Table 1:** Examples of information provided by two breeding companies about live body mass development of their chicken strains for egg laying.

Strain	Production Purpose	Average Body Mass in kg (range) at Week 20 i.e., Onset of Laying	Average Body Mass in kg (Range) at End of Laying Period	Relative Growth after Onset of Laying i.e., Week 20 – End of Laying Period	Reference
Lohmann LSL-Classic	egg laying	1.385 (1.33–1.44)	1.80 (1.70–1.90)	29.96%	[20]
Lohmann Brown-Classic	egg laying	1.65 (1.60–1.70)	2.05 (1.90–2.20)	24.24%	[21]

**Table 2:** Examples of information provided by two breeding companies about live body mass development of their chicken strains for meat production.

Strain	Production Purpose	Average Hatching Body Mass in g	Average Body Mass in kg at Production End (Day 35 Assumed)	Relative Growth during Fattening Period	Reference
ROSS 308 Broiler	meat production	42	2.144	5004.76%	[22]

## Supplementary Materials

The following are available online at https://www.mdpi.com/2076-2615/9/6/348/s1, Table S1: Calculated FSR’s (Floor space requirement) based on Equations (2a–c) and the respective average FSRX. The table only shows two decimals places, calculations were conducted with exact numbers, Table S2: Additional space for layer pullets when applying the Directives (1999/74/EC) for adult laying hens and (2007/43/EC) for broiler chicken. The table only shows one decimal place, but calculations were conducted with exact numbers. Data with * were omitted from the respective Figure 1a as they show negative values and we chose for graphical reasons to start the y-axis at zero, Table S3: Relative additional space when applying the Council Directives(1999/74/EC) for adult laying hens and (2007/43/EC) for broiler chickens. The table only shows three decimals places, calculations, but were conducted with exact numbers. Data with * were omitted from the respective Figure 1b as they show negative values and we chose for graphical reasons to start the y-axis at zero, Equation (S1): Calculation of how a relative additional space of 60% can be transferred into a stocking density parameter with birds/area, Equation (S2): Calculation of how a relative additional space of 20% can be transferred into a stocking density parameter with birds/area.
