# Supplementary material for: Suggestions to Derive Maximum Stocking Densities for Layer Pullets
Source: Animals (Basel). 2019 Jun 13;9(6):348. doi: 10.3390/ani9060348 (PMC6617375; doi:10.3390/ani9060348)
Supplement: Supplementary file 1 [file animals-09-00348-s001.pdf]

Table S1. *Calculated FSR (Floor space requirement) based on equations 2a-c and the respective average FSR_X. The table only shows two decimals places, calculations were conducted with exact numbers.*

Live body mass of pullet in g	FSR_{LT} in cm^2	FSR_{LB} in cm^2	FSR_{LSL} r in cm^2	**average FSR_x in cm^2**
100	129,3	144,5	111,2	**128,3**
200	152,3	164,8	133,7	**150,3**
300	175,3	185,1	156,2	**172,2**
400	198,3	205,4	178,6	**194,1**
500	221,3	225,7	201,1	**216,1**
600	244,4	246,0	223,6	**238,0**
700	267,4	266,3	246,1	**259,9**
800	290,4	286,6	268,5	**281,9**
900	313,4	306,9	291,0	**303,8**
1000	336,4	327,3	313,5	**325,7**
1100	359,5	347,6	335,9	**347,7**
1200	382,5	367,9	358,4	**369,6**
1300	405,5	388,2	380,9	**391,5**
1400	428,5	408,5	403,3	**413,5**
1500	451,5	428,8	425,8	**435,4**
1600	474,6	449,1	448,3	**457,3**

***Table S2**. Additional space for layer pullets when applying the Directives (1999/74/EC) for adult laying hens and (2007/43/EC) for broiler chicken for meat production. The table only shows one decimal place, but calculations were conducted with exact numbers. Data with * were omitted from the respective Figure 2a as they show negative values and we chose for graphical reasons to start the y-axis at zero.*

**Body mass of pullet in g**	**additional space in cm² when applying (1999/74/EC)**	**additional space in cm² when applying (2007/43/EG; 33 kg/m²)**	**additional space in cm² when applying (2007/43/EG; 39 kg/m²)**	**additional space in cm² when applying (2007/43/EG; 42 kg/m²)**
100	982,7	-103,2*	-107,8*	-109,7*
200	960,8	-83,3*	-92,6*	-96,3*
300	938,8	-63,4*	-77,4*	-82,9*
400	916,9	-43,5*	-62,2*	-69,5*
500	895,0	-23,6*	-46,9*	-56,1*
600	873,0	-3,8*	-31,7*	-42,7*
700	851,1	16,0	-16,5*	-29,3*
800	829,2	35,9	-1,3*	-15,9*
900	807,2	55,8	13,8	-2,5*
1000	785,3	75,7	29,1	10,7
1100	763,4	95,6	44,3	24,1
1200	741,4	115,4	59,5	37,5
1300	719,5	135,3	74,7	50,9
1400	697,6	155,2	89,9	64,3
1500	675,6	175,1	105,2	77,7
1600	653,7	195,0	120,4	91,1

Table S3. *Relative additional space when applying the Council Directives (1999/74/EC) for adult laying hens and (2007/43/EC) for chickens for meat production. The table only shows three decimals places, calculations, but were conducted with exact numbers. Data with * were omitted from the respective Figure 2b as they show negative values and we chose for graphical reasons to start the y-axis at zero.*

**live body mass in g**	relative **additional space in cm^2 when applying (1999/74/EC)**	relative **additional space in cm^2 when applying** (2007/43/EC; 33 kg/m2)	relative **additional space in cm^2 when applying** (2007/43/EC; 39 kg/m2)	relative **additional space in cm^2 when applying** (2007/43/EC; 42 kg/m2)
100	0,884	-3,406*	-4,207*	-4,607*
200	0,864	-1,375*	-1,806*	-2,022*
300	0,844	-0,697*	-1,006*	-1,161*
400	0,825	-0,359*	-0,606*	-0,730*
500	0,805	-0,156*	-0,366*	-0,471*
600	0,785	-0,020*	-0,206*	-0,299*
700	0,766	0,075	-0,092*	-0,176*
800	0,746	0,148	-0,006*	-0,083*
900	0,726	0,204	0,060	-0,012*
1000	0,706	0,249	0,113	0,045
1100	0,687	0,286	0,157	0,092
1200	0,667	0,317	0,193	0,131
1300	0,647	0,343	0,2243	0,164
1400	0,627	0,365	0,250	0,193
1500	0,608	0,385	0,273	0,217
1600	0,588	0,402	0,293	0,239

Equation S1: *Calculation of how a relative additional space of 60% can be transferred into a stocking density parameter with birds/area.*

$$(S1a)\ rel.add.space\ (R) = \frac{additional\ space\ (Z)}{additional\ space\ (Z) + space\ covered\ by\ body\ (FSR)}$$

$$(S1b) \Rightarrow \frac{6}{10} = \frac{Z}{Z + FSR}$$

$$(S1c) \Rightarrow \frac{6}{10}Z + \frac{6}{10}FSR = Z$$

$$(S1d) \Rightarrow\ 6Z + 6FSR = 10Z$$

$$(S1e) \Rightarrow\ 6FSR = 4Z$$

$$(S1f) \Rightarrow \frac{6}{4}FSR = Z$$

$$(S1g) \Rightarrow \frac{3}{2}\ FSR = Z$$

Equation S2*: Calculation of how a relative additional space of 20% can be transferred into a stocking density parameter with birds/area.*

$$(S2a)\ rel.add.space\ (R) = \frac{additional\ space\ (Z)}{additional\ space\ (Z) + space\ covered\ by\ body\ (FSR)}$$

$$(S2b) \Rightarrow \frac{2}{10} = \frac{Z}{Z + FSR}$$

$$(S2c) \Rightarrow \frac{2}{10}Z + \frac{2}{10}FSR = Z$$

$$(S2d) \Rightarrow\ 2Z + 2FSR = 10Z$$

$$(S2e) \Rightarrow\ 2FSR = 8Z$$

$$(S2f) \Rightarrow \frac{2}{8}FSR = Z$$

$$(S2g) \Rightarrow \frac{1}{4}\ FSR = Z$$
